# Anti-Inflammatory and Anti-Allergic Activities of Pentaherb Formula, Moutan Cortex (Danpi) and Gallic Acid

**DOI:** 10.3390/molecules18032483

**Published:** 2013-02-25

**Authors:** Kelly Y. P. Liu, Shuiqing Hu, Ben C. L. Chan, Elaine C. L. Wat, Clara B. S. Lau, Kam L. Hon, Kwok P. Fung, Ping C. Leung, Patrick C. L. Hui, Christopher W. K. Lam, Chun K. Wong

**Affiliations:** 1 Department of Chemical Pathology, the Chinese University of Hong Kong, Prince of Wales Hospital, Shatin, NT, Hong Kong; 2 Institute of Chinese Medicine, the Chinese University of Hong Kong, Hong Kong; 3 State Key Laboratory of Phytochemistry and Plant Resources in West China, the Chinese University of Hong Kong, Hong Kong; 4 Department of Paediatrics, the Chinese University of Hong Kong, Prince of Wales Hospital, Shatin, NT, Hong Kong; 5 School of Biomedical Sciences, Faculty of Medicine, the Chinese University of Hong Kong, Hong Kong; 6 Institute of Textiles and Clothing, the Hong Kong Polytechnic University, Hung Hom, Kowloon, Hong Kong; 7 State Key Laboratory of Quality Research in Chinese Medicine, Macau Institute for Applied Research in Medicine and Health, Macau University of Science and Technology, Macau

**Keywords:** anti-allergic, anti-inflammatory, gallic acid, moutan cortex, pentaherb formula

## Abstract

Pentaherb formula (PHF) has been proven to improve the quality of life of children with atopic dermatitis without side effects. The aim of this study was to elucidate the potential anti-inflammatory and anti-allergic activities of PHF, Moutan Cortex (Danpi/DP) and gallic acid (GA) using human basophils (KU812 cells), which are crucial effector cells in allergic inflammation. PHF, DP and GA could significantly suppress the expression of allergic inflammatory cytokine IL-33-upregulated intercellular adhesion molecule (ICAM)-1, and the release of chemokines CCL2, CCL5, CXCL8 and inflammatory cytokine IL-6 from KU812 cells (all *p* < 0.05). With the combined use of dexamethasone (0.01 μg/mL) and GA (10 μg/mL), the suppression of ICAM-1 expression and CCL5 and IL-6 release of IL-33-activated KU812 cells were significantly greater than the use of GA alone (all *p* < 0.05). The suppression of the IL-33-induced activation of intracellular signalling molecules p38 mitogen activated protein kinase, nuclear factor-κB and c-Jun amino-terminal kinase in GA-treated KU812 cells could be the underlying mechanism for the suppression on ICAM-1, chemokines and cytokines. The combined use of dexamethasone with the natural products PHF or DP or GA might therefore enhance the development of a novel therapeutic modality for allergic inflammatory diseases with high potency and fewer side effects.

## 1. Introduction

The prevalence of allergic diseases such as allergic rhinitis, asthma and atopic dermatitis (AD) has been increasing dramatically in both developed and developing countries [1,2,3]. The World Health Organization (WHO) estimated that in 2011, 300 million people in the World population of 7.1 billion (about 4%) have allergic asthma, adversely affecting their quality of life and the socio-economic welfare of the society [3]. AD is one of the most frequent chronic inflammatory skin diseases, affecting up to 25% of children and 1–3% of adults worldwide [1]. Also named eczema, AD is the most common type of chronic allergic skin diseases. It can occur at any age, affecting infants, children and adults. The worldwide prevalence of AD is increasing with about 70% of cases occurring before the age of 5 [4]. AD infants are prone to subsequently develop allergic asthma and allergic rhinitis during childhood [3,5,6].

AD is a long-lasting skin disorder; patients may suffer from episodic exacerbations and remissions during their life [7]. Typical symptoms of AD include extremely itchy, inflamed and dry skin, the inflamed area can be red, swollen, cracked, scaled, webby and crusted [7,8]. There is no definitive cure for AD and the current effective treatment involves topical application of immunosuppressive steroids with undesirable side effects. Therefore, there has been a rising interest in the development of safer and non-steroid immunomodulatory formulas for the treatment of AD. Traditional Chinese Medicine (TCM) has become a more accepted and increasingly used modality for immunomodulation, especially in Asia. 

Our previous clinical trial has shown that children with moderate-to-severe AD manifested significantly improved quality of life after a 12-week treatment with TCM Pentaherb formula (PHF) concurrently with reduced use of topical corticosteroids [9]. Such a finding suggested that PHF could potentially be an alternative adjunct therapy for AD. PHF contains five herbs: Lonicerae Flos (Jinyinhua), Menthae Herba (Bohe), Moutan Cortex (Danpi/DP), Atractylodis Rhizoma (Cangzhu), and Phellodendri Cortex (Huangbai) in a ratio of 2:1:2:2:2 [9]. Our group and others have previously shown that different herbs in PHF possessed anti-inflammatory activities and exhibit potential therapeutic efficacy on inflammatory diseases such as rheumatoid arthritis [10,11,12]. Therefore, PHF has been formulated to further evaluate its cellular mechanisms for anti-inflammatory activities. PHF with an increased ratio of Danpi exhibited a greater inhibitory effect on the release of the cytokines interleukin (IL)-6 and IL-1 from HMC-1 human mast cells [13]. Gallic acid (GA, 3,4,5-trihydroxybenzoic acid), a potent anti-oxidant found in witch hazel and tea leaves, is one of the main ingredients in Danpi [14]. GA has been shown to possess anti-tumorigenic effect and anti-inflammatory activity in both nude mice and human cell lines [15,16,17,18,19].

IL-33 is a novel member of the IL-1 cytokine family that is released passively during cell necrosis and tissue damage [20,21,22,23]. It has been characterized as a potent pro-inflammatory Th2 cytokine that acts on immune cells such as mast cells, eosinophils and basophils in allergic inflammation [20,21,22,23]. In addition, the inflamed skin lesions of AD patients have both elevated protein and mRNA expression of IL-33 compared to normal subjects [20,21,22,23].

Tissue inflammation consists of two essential steps: the adherence of leukocytes to vascular endothelial cells before their migration to inflammatory sites via diapedesis, and release of allergic inflammatory chemokines and cytokines by the leukocytes [24,25,26]. The aim of the present study was to elucidate the mechanisms of the *in vitro* anti-inflammatory and anti-allergic activities of PHF, Danpi and GA via their modulation of: (i) expression of cell surface adhesion molecules and (ii) the release of chemokines and cytokines from allergy-related alarmin IL-33-activated human basophils, which are crucial effector cells of allergic inflammation in allergic asthma and AD [27]. Circulating basophils can be recruited to the inflammatory tissues in allergic disorders including allergic asthma, AD and allergic rhinitis [28]. During asthma exacerbation and in response to allergen inhalation challenge, basophils markedly infiltrate into allergic inflammatory sites [29]. Since human basophils represent 1% of peripheral blood leukocytes and only 2.5 × 10^6^ cells can be purified from 4 × 10^8^ peripheral blood mononuclear cells, it is not feasible to obtain enough number of basophils for the present *in vitro* studies. According to our previous experiments, we found that the representative KU812 basophilic cells showed similar results for the expression of ICAM-1 and induction of chemokines and cytokines (data not shown). Therefore, like in our previous publication, we adopted the representative KU812 basophilic cells for the present mechanistic study [27]. 

## 2. Results and Discussion

### 2.1. Effect of PHF, Danpi and GA on Cell Surface Expression of Adhesion Molecule

**Figure 1:**
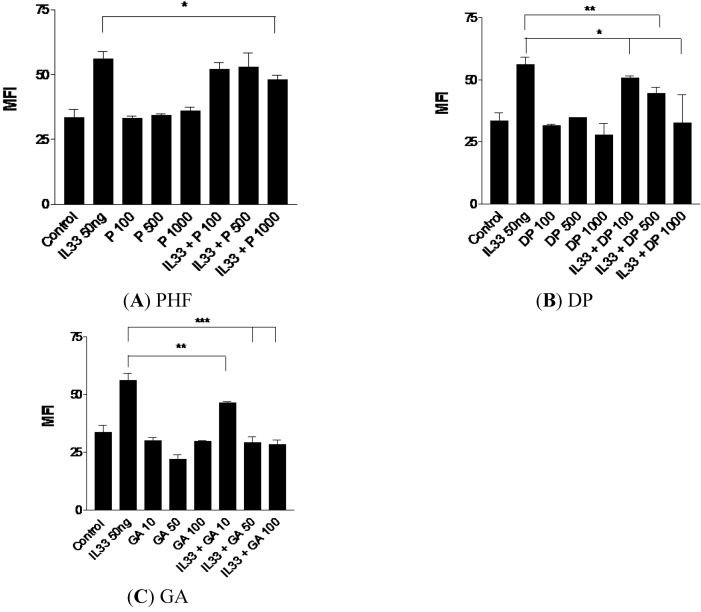
Suppressive effect of ICAM-1 expression on IL-33-activated human basophilic KU812 cells treated with (**A**) PHF (100, 500 and 1,000 μg/mL), (**B**) DP (100, 500 and 1,000 μg/mL), (**C**) GA (10, 50 and 100 μg/mL) for 18 h. As shown in bar charts, surface expressions of ICAM-1 on 5,000 KU812 cells are expressed as the mean plus SEM of MFI and normalized by subtracting appropriate isotypic control of three independent experiments. * *p* < 0.05, ** *p* < 0.01, *** *p* < 0.005. P100-1000: PHF 100–1,000 μg/mL, DP100-1000: Danpi 100–1,000 μg/mL, GA10-100: GA 10–100 μg/mL.

IL-33-activated KU812 cells were treated with various concentrations of PHF, DP and GA, followed by measurement of cell surface expression of adhesion molecule. As shown in Figure 1, PHF, DP and GA could significantly suppress the expression of IL-33-induced intercellular adhesion molecule (ICAM)-1 on KU812 cells in a dose-dependent manner (all *p* < 0.05). The reduced ICAM-1 expression on IL-33 activated KU812 cells suggests that PHF, DP and GA may inhibit the endothelial transmigration of basophiles to the inflamed sites, and dampen the subsequent allergic responses [30,31,32].

### 2.2. Effect of PHF, DP and GA on Inflammation-Related Chemokines CCL2, CCL5 and CXCL8 Production from IL-33-Activated KU812 Cells

Chemokine CCL2 (monocyte chemotactic protein-1/MCP-1), CCL5 (regulated and normal T cell expressed and secreted/RANTES) and CXCL8 (IL-8) are known to be associated with inflammation [33,34,35,36]. CCL2 is known to be an AD-associated chemokine that recruits dendritic cells, monocytes and memory T cells from the circulation to inflammatory sites [33]. CXCL8 is a potent chemo-attractant for immune cells, especially neutrophils, to the inflamed sites where CCL5 is chemotactic for T helper type 2 (Th2) cells, eosinophils and basophils [33,34,35,36].

**Figure 2:**
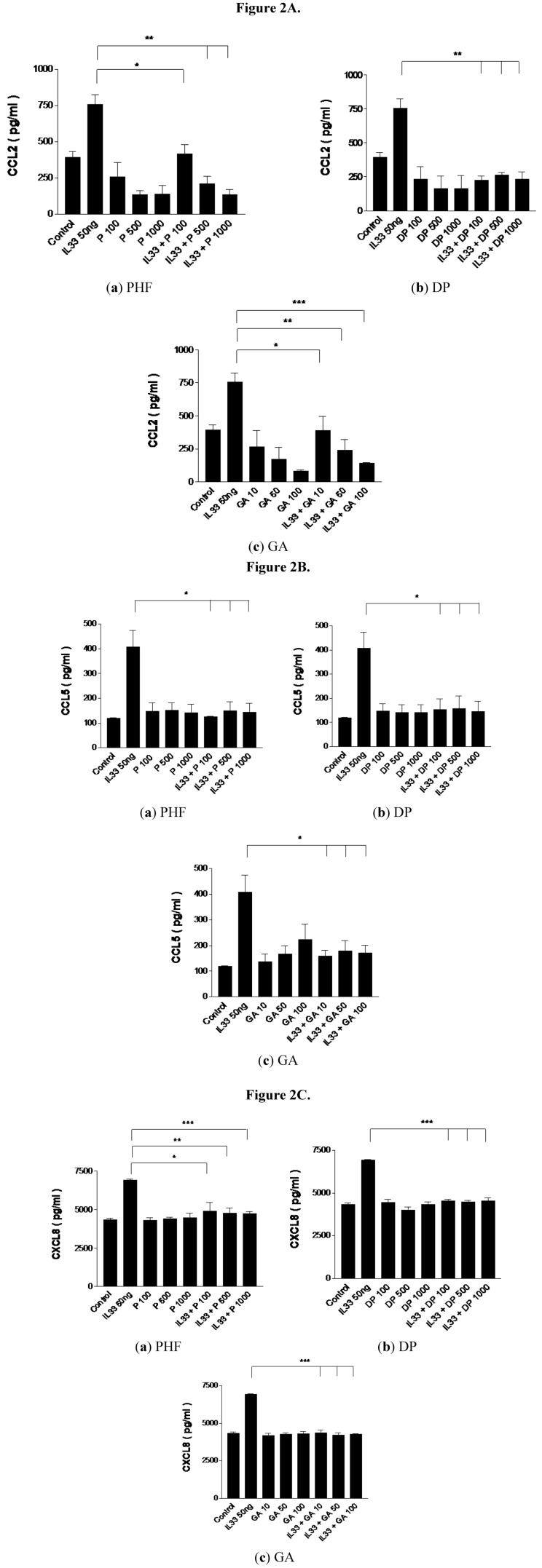
Suppressive effect on (**A**) CCL2, (**B**) CCL5 and (**C**) CXCL8 release from IL-33-activated human basophilic KU812 cells treated with PHF (100, 500 and 1,000 μg/mL), DP (100, 500 and 1,000 μg/mL) or GA (10, 50 and 100 μg/mL) for 18 h. Release of chemokines in culture supernatants was determined by CBA. * *p* < 0.05, ** *p* < 0.005, *** *p* < 0.001. P100-1000: PHF 100–1,000 μg/mL, DP100-1000: Danpi 100–1,000 μg/mL, GA10-100: GA10–100 μg/mL.

Figure 2 shows that the release of CCL2, CCL5 and CXCL8 from IL-33-activated KU812 cells was significantly suppressed by the treatment with PHF, DP and GA. There was a dose-dependent suppression of CCL2 release by PHF and GA (Figure 2A). The suppressed release of these inflammation-related chemokines from IL-33-activated KU812 cells may reduce the infiltration of immune cells such as Th cells, eosinophils, basophils and macrophages to the inflamed sites, thereby lessening the subsequent inflammation and other allergic responses. Th cells including Th1 and Th2 which play important roles for the cell-mediated immunity and humoral immunity, respectively [35]. Since Th2 cells is responsible for the IgE production for the sensitization and activation of mast cells in type I hypersensitivity, the suppression of the release of Th2 chemokine CCL5 by PHF, DP and GA can subsequently reduce the infiltration of Th2 cells into the inflammatory site and hence the allergic inflammation [34,35].

### 2.3. Effect of PHF, DP and GA on Inflammatory Cytokine IL-6 Production from IL-33-Activated KU812 Cells

**Figure 3:**
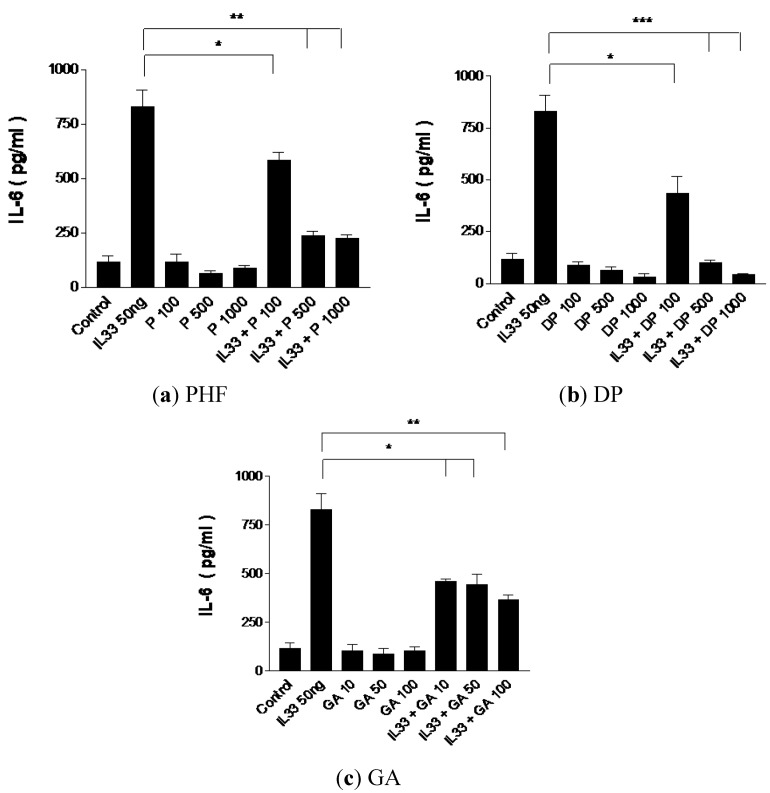
Suppressive effect of inflammatory cytokine IL-6 release from IL-33-activated human basophilic KU812 cells treated with PHF (100, 500 and 1,000 μg/mL), DP (100, 500 and 1,000 μg/mL) or GA (10 and 100 μg/mL) for 18 h. Release of IL-6 in culture supernatants was determined by CBA. * *p* < 0.05, ** *p* < 0.005, *** *p* < 0.001. P100-1000: PHF 100–1,000 μg/mL, DP100-1000: DP 100–1,000 μg/mL, GA10-100: GA10–100 μg/mL.

The proinflammatory cytokine IL-6 is produced by T cells and macrophages at the inflammatory site. It plays an important role in the regulation of immune responses, immediate and late-phase allergic inflammation, and Th17 cell activation [37,38,39]. The combined effects of IL-6 with soluble IL-6 receptor-α can lead to the transition of acute to chronic inflammation by means of switching transmigration from neutrophils to monocytes/macrophages into the inflamed site [37,38,39]. As shown in Figure 3, the dose-dependent suppression of IL-6 release by PHF, DP and GA from IL-33-activated KU812 cells may reduce the progression from acute to chronic inflammation in allergic diseases. 

### 2.4. Effect of GA on the Phosphorylation of Intracellular Signaling Molecules p38 Mitogen Activated Protein Kinase (MAPK), Inhibitory-κBα (IκBα) and c-Jun Amino-terminal Kinase (JNK) on IL-33 Activated KU812 Cells

**Figure 4:**
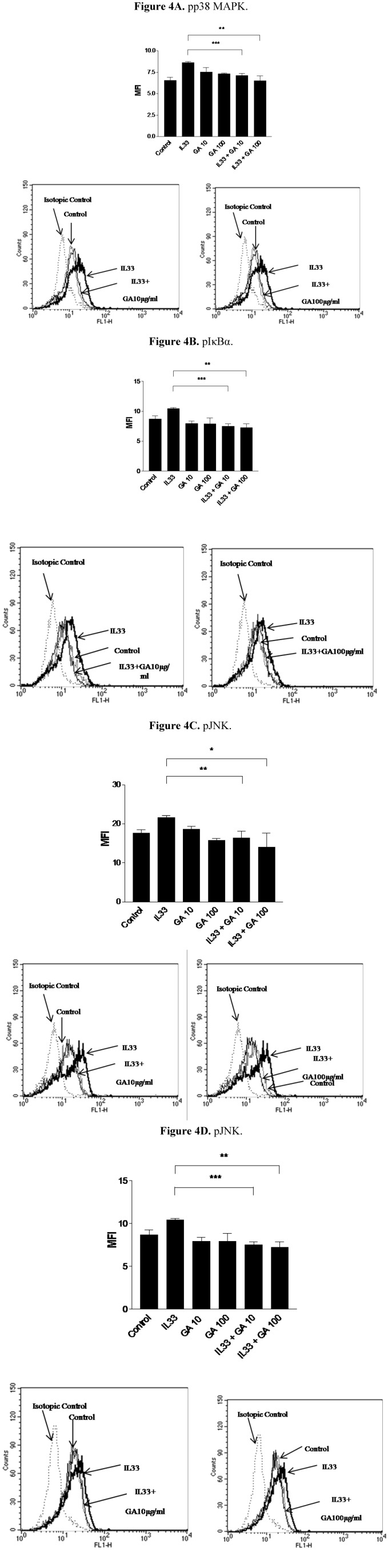
Suppressive effects of IL-33-induced-intracellular signaling molecules by 15-min treatment with GA in KU812 cells. (**A**) phosopho (p) p38 MAPK pretreated with IL-33 (30 min); (**B**) pIκBα_pretreated with IL-33 (1 h); (**C**) pJNK pre-treated with IL-33 (30 min) and (**D**) pJNK pretreated with IL-33 (1 h). Results are shown as the mean plus SEM of MFI and normalized by subtracting appropriate isotypic control of three independent experiments in bar charts. Representative histograms of intracellular expression of phosphor-signaling molecules in KU812 cells were also shown. * *p* < 0.05, ** *p* < 0.005, *** *p* < 0.001. P100-1000: PHF 100–1,000 μg/mL, DP100-1000: DP 100–1,000 μg/mL, GA10-100: GA10–100 μg/mL.

p38 MAPK, NF-κB and JNK are involved in the intracellular signaling pathways contributing to the inflammatory responses in granulocytes such as basophils and eosinophils [20,27,40]. Both p38 MAPK and JNK play crucial roles in regulating cell apoptosis, cell proliferation and inflammatory cytokines release [41,42,43,44]. NF-κB, bound by inhibitory protein IκBα, is a key regulator of pro-inflammatory mediator expression and inflammatory cytokines induction in lymphocytes, granulocytes, macrophages and fibroblasts [20,40,41,42,43,44]. The level of the phosphorylation of IκBα is directly related to the release of active NF-κB and the subsequent translocation into the nucleus to activate the genes transcription for cytokines, chemokines and adhesion molecules. Figure 4 shows that the IL-33-upregulated phosphorylation of p38 MAPK, IκBα and JNK, in KU812 cells was significantly inhibited by GA (all *p* < 0.05). However, GA did not exhibit any effect on the phosphorylation of extracellular signal regulated kinase (ERK) (data not shown). Therefore, the reduction of the phosphorylation of p38 MAPK, IκBα and JNK by GA could be the underlying intracellular mechanisms accounting for the suppression of ICAM-1 expression and release of CCL2, CCL5, CXCL8 and IL-6. In Figure 4A, since GA at dose 10 μM could potently suppress the IL-33-induced phosphorylation of p38MAPK, GA (100 μM) did not show any significant further suppression on the IL-33-induced phosphorylation of p38MAPK (*p* > 0.05). However, the mean of the phosphorylation of p38MAPK of GA (100 μM) is still lower than that of GA (10 μM). Since PHF and DP are complex heterogeneous mixtures but not single and purified compound, it could be difficult to interpret their effect on the individual intracellular signaling pathways. Therefore, PHF and Danpi were not investigated for their effects on intracellular signaling molecules in the present study. 

### 2.5. Enhanced Suppressive Effect on ICAM-1, CCL5 and IL-6 Expression of IL-33-Activated KU812 Cells with the Combined Treatment of Dexamethasone and GA

Dexamethasone, a potent synthetic member of the glucocorticoid class of steroid drugs, is usually prescribed for patients with AD for resolving the tissue inflammation. It has been reported that dexamethasone can promote eosinophilic apoptosis [45] and inhibit basophilic migration [46]. 

**Figure 5:**
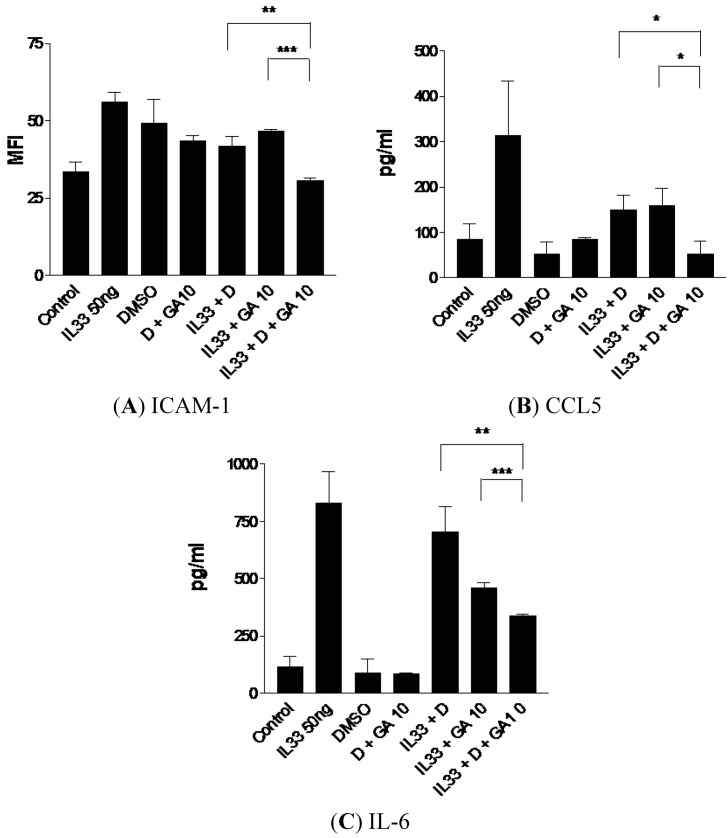
Suppressive effects of (**A**) ICAM-1 expression, (**B**) CCL5 and (**C**) IL-6 release of IL-33-activated human basophilic KU812 cells treated with dexamethasone (0.01 μg/mL) with or without GA (10 μg/mL) together. (**A**) Surface expressions of ICAM-1 on 5,000 KU812 cells are expressed as the mean plus SEM of MFI and normalized by subtracting appropriate isotypic control of three independent experiments. (**B**, **C**) Release of CCL5 and IL-6 in culture supernatants was determined by CBA. * *p* < 0.05, ** *p* < 0.005, *** *p* < 0.001. D: Dexamethasone 0.01 μg/mL, DMSO: dimethyl sulfoxide 0.2% (vol/vol), GA10: GA 10 μg/mL, GA100: GA 100 μg/mL.

We investigated the expression of adhesion molecules, chemokines and cytokines in IL-33-activated KU812 cells treated with the combined use of dexamethasone at various concentrations (0.01, 0.1 and 1 μg/mL) and the 3 natural products (PHF, DP and GA). As shown in Figure 5, the combined use of a low concentration of dexamethasone (0.01 μg/mL) with GA (10 μg/mL) could further suppress ICAM-1, CCL5 and IL-6 expression of KU812 cells compared to the use of GA (10 μg/mL) or dexamethasone (0.01 μg/mL) alone. 

However, higher concentrations of dexamethasone (0.1 and 1 μg/mL) together with GA (10 μg/mL) did not exhibit any significant suppression of ICAM-1, CCL5 and IL-6 comparing with GA or dexamethasone alone (data not shown). In addition, PHF and DP (100 μg/mL) showed similar enhanced suppressive effect on the IL-33-induced expression of ICAM-1, CCL5 and IL-6 (data not shown). The above results suggest that PHF, DP or GA could be used together with a lower dose of dexamethasone for the resolution of inflammation in patients with allergic diseases such as AD, thereby promoting the therapeutic efficacy and lessening the adverse side effect of steroidal drugs. 

## 3. Experimental

### 3.1. Reagents

RPMI-1640 medium and fetal bovine serum (FBS) were purchased from Life Technology Corp. (Carlsbad, CA, USA), while FITC conjugated goat anti-mouse IgG, dexamethasone, PBS, dimethyl sulfoxide (DMSO) and GA were purchased from Sigma-Aldrich Co. (St. Louis, MO, USA). Recombinant human IL-33, FITC-conjugated mouse anti-human ICAM-1/CD54, anti-human L-selectin/CD62 monoclonal antibody were purchased from R&D Systems Inc. (Minneapolis, MN, USA). Purified mouse IgG1κ isotype control, mouse anti-human IκBa (pS32/pS36), mouse anti-JNK (pT183/pY185), mouse anti-p38MAPK (pT180/pY182) were purchased from BD Pharmingen Corp., (San Diego, CA, USA). 

### 3.2. Preparation of Plant Extracts

PHF and DP were supplied by the Institute of Chinese Medicine, The Chinese University of Hong Kong, from which water extract was prepared by the Hong Kong Biotechnology Institute, Hong Kong. For PHF extract, Lonicerae Flos (20 g), Menthae Herba (10 g), Moutan Cortex (20 g), Atractylodis Rhizoma (20 g) and Phellodendri Cortex (20 g) were refluxed in distilled water (900 mL) of at 100 °C for 2 h and the whole process was repeated twice. The three batches of water extracts were mixed together and centrifuged to remove the herbal debris. Finally, the combined extract was vacuum dried to form herbal powder which was stored in desiccators until use. These processes fulfilled the Good Manufacturing Practice according to the Australian Therapeutic Goods Administration standard. GA was purchased from Sigma-Aldrich. The dosages of DP used in this study were 100–1,000 µg/mL. Based on our previous high-performance liquid chromatography (HPLC) analysis, the amount of GA present in DP was 3.5% w/w. Hence, the dosages of GA used for the experiments were in the range of 10–100 µg/mL. The PHF, Danpi and GA used in this *in vitro* study were weighed and dissolved in pyrogen-free phosphate buffered saline (PBS) at 40 °C for 4 h, centrifuged at 2,000 rpm for 5 min. The supernatants were filtered through 0.22 μm polyethersulphone filter. If there was any herbal debris, it was dried at 57 °C overnight for weighing. The solutions were stored at 4 °C. The concentration of the solution = (weighted mass before dissolving in PBS − mass of herbal debris) / volume of PBS used. 

### 3.3. HPLC Analyses of GA and Danpi Aqueous Extract

HPLC analyses were performed using Hewlett Packard Agilent 1100 series HPLC System, equipped with G1329A ALS Autosampler and G1315A Diode Array Detector (Agilent Technologies, Santa Clara, CA, USA). Calibration curves for GA was prepared using standard solutions containing 0, 5, 10, 15, and 20 μg/mL GA. Danpi aqueous extract was prepared with double distilled water (800 μg/mL). Sample solution was injected onto an Ultrasphere ODS column (250 × 4.6 mm i.d., particle size 5 μm, Beckman Instrument Inc., Fullerton, CA, USA). All solvents were pre-filtered with 0.45 μm Millipore filter disk (Millipore Corp., Billerica, MA, USA) and de-gassed. A gradient elution was carried out using the following solvent systems: mobile phase A—acetonitrile; mobile phase B—double distilled water/phosphoric acid (99.0/1.0; v/v). The analyses were performed for 55 min. The flow rate used was 1.0 mL/min and detection was performed at 274 nm. Each sample (10 μL) was injected into the column after filtration through a 0.45 μm filter disk. The system was monitored by a computer equipped with the 32 Karat Software (Beckman) for data collection, integration and analysis of GA quantity. 

### 3.4. Cell Culture and Treatment

The human basophilic cell line KU812 cells (American Type Culture Collection, Manassas, VA, USA) were maintained in RPMI-1640 medium supplemented with 10% (v/v) FBS (RPMI-1640 complete medium). Cells were incubated at 37 °C in atmospheric air enriched with 5% (v/v) CO_2_. At 70–80% cell confluence, cells dispersed in RPMI-1640 complete medium to a final cell concentration of 10 × 10^5^ cells/ml for further treatment. 

### 3.5. Immunofluorescence Staining and Flow Cytometry for the Analysis of ICAM-1, Cytokines and Intracellular Signaling Molecules

KU812 cells were seeded in 24-well plate at a density of 5 × 10^5^ cells in 500 μL RPMI-1640 complete medium per well. They were then pre-activated with IL-33 (50 ng) for 1 h followed by treatment with various concentrations of PHF, DP or GA in the presence of IL-33 for further 18 h. Cells were then used for the detection of expression of adhesion molecules, while the supernatants collected was subjected to BD cytometric beads array (CBA) assays for allergic inflammation-related chemokines and cytokine (see below) using flow cytometry.

For the analysis of adhesion molecules, cells were washed and suspended in cold PBS followed by blocking with 2% human pooled serum for 20 min at 4 °C. They were incubated with FITC-conjugated mouse anti-human ICAM-1 or mouse IgG1 isotype (R&D Systems) for 40 min at 4 °C in the dark. After washing, the cells were re-suspended in PBS for flow cytometric analysis.

For the analysis of chemokines and cytokines, supernatants obtained were subjected for the bead-based multiplex CBA immunoassay with Human Inflammatory Kit (CXCL8, IL-1β, IL-6, IL-10, TNF-α and IL-12p70) and Human Chemokine Kit (CXCL8, CCL5, CXCL9, CCL2 and CXCL10). The capture beads contained monoclonal antibodies against individual cytokine and chemokine. Supernatant (50 μL) was incubated with different capture bead mixtures (50 μL) and phycoerythrin-conjugated detection antibodies (50 μL) for three hours at room temperature with constant shaking. After incubation, the beads were washed and re-suspended in wash buffer. The samples were subsequently analyzed by BD FACSCalibur flow cytometer (BD Biosciences, San Jose, CA, USA) with BD CBA analysis software.

For the analysis of intracellular expression of phosphorylated signaling molecules, 5 × 10^5^ cells seeded in 24-well culture plate were pre-activated with IL-33 (50 ng) for 30 min or 1 h followed by 15-min treatment with GA. After cells were fixed with 4% paraformaldehyde for 10 min at 37 °C and centrifugation, cells were permeabilized in ice-cold methanol for 30 min and then stained with FITC-conjugated mouse anti-human phosphorylated ERK1/2, phosphorylated JNK, phosphorylated p38 MAPK, phosphorylated IκBα or mouse IgG1 antibodies (BD Pharmingen) for 30 min at 4 °C in the dark. Cells were then washed, resuspended and subjected to analysis.

Expressions of surface adhesion molecule, intracellular phosphorylated signaling molecules and chemokines and cytokines were analyzed by flow cytometry (FACSCalibur) as mean fluorescence intensity (MFI), which includes both the changes of target molecule expression in individual cells and the percentage of cells expressing the target molecules.

### 3.6. Statistical Analysis

All data are expressed as the means ± SEM. Differences between groups were assessed by Students’s t-test. A probability *p* < 0.05 was considered significantly different. When ANOVA indicated a significant difference, the Bonferroni post hoc test was then used to assess the difference between groups. All analyses were performed using the Statistical Package for the Social Sciences statistical software for Windows, version 16.0 (SPSS Inc., Chicago, IL, USA).

## 4. Conclusions

Since the immunopathological mechanism of allergic diseases such as AD is complex, involving both cellular and humoral components of the immune system [47], the above results suggest that TCM Pentaherb Formula and its herbal component Danpi and bioactive compound gallic acid can suppress inflammation elicited by IL-33-activated allergic inflammation-related effector cells, namely, the basophils, by the inhibition of the expression of adhesion molecules and allergy-related cytokines and chemokine. Our present study has also provided evidence for the intracellular mechanisms including the MPAK and NF-κB pathways by which GA could modulate the function of basophils. Since corticosteroids are not present in Pentaherb Formula [48], the combined use of lower dose of dexamethasone with natural product Pentaherb Formula or Danpi or gallic acid may enhance the development of the novel therapeutic modality for allergic inflammatory diseases such as AD with high potency and less side effects.
